# The wound healing action of a cream latex formulation of *Jatropha gaumeri* Greenm. in a pre-clinical model

**DOI:** 10.14202/vetworld.2020.2508-2514

**Published:** 2020-11-25

**Authors:** Floribeth León, Vianey Hernandez-Zapata, Manuel Chan Bacab, Guadalupe Maldonado, Juan Arana Lezama, Victor Monteon

**Affiliations:** 1 Facultad Odonttología, Universidad Autonoma Campeche, Mexico; 2 Departamento Biotecnología, Universidad Autonoma Campeche, Mexico; 3 Centro Investigaciones Biomedicas, Universidad Autonoma Campeche, Mexico; 4 Hospital General de Especialidades, Campeche, Mexico

**Keywords:** alkaloids, *Jatropha gaumeri* latex, wound healing

## Abstract

**Background and Aim:**

*Jatropha gaumeri* Greenm. is commonly used to treat mouth blisters and skin rashes, its latex has analgesic and anti-inflammatory activity on buccal ulcer. This study aimed to demonstrate the wound healing activity of a cream formulation of *Jatropha gaumeri* Greenm. latex in a murine model, provide a histological assessment of its scarring effects, and identify the family of phytochemicals involved in these effects.

**Materials and Methods:**

Latex was obtained from the cut stalk leaves and young stems of *J. gaumeri* and stored in sterile tubes with protection from light. Chloroform, ethyl acetate, and aqueous fractions of the latex were obtained. Fifty male Balb/c mice aged 10-12 weeks were divided into10 groups of five mice: Group 1 corresponded to healthy mice with wounds; Group 2 corresponded to mice with wounds and treated with A-Derma^®^; and from Group 3 to group 10 corresponded to mice treated with a different latex fraction. A circular skin wound of about 1 cm was made on the paravertebral region of each mouse under anesthetized and aseptic conditions. The wounds were topically treated every 24 h with the respective extracts for 22 days, after which skin tissue specimens were obtained and stained with hematoxylin-eosin and Masson’s trichrome. The efficiency of healing was measured by quantifying the tensile strength of the scars. The phytochemicals in the latex were elucidated using thin chromatography.

**Results:**

The aqueous latex fraction produced the best wound healing activity and was superior to the positive control. Reepithelialization at the histological level resulted in tissue that resembled healthy skin in terms of the appearance of collagen, the regeneration of hair follicles, and cellularity of the dermis, which showed organized epithelialization. A wound healing efficacy of 97% was observed, and it seems that alkaloids were the phytochemicals mostly likely responsible for these effects.

**Conclusion:**

*J. gaumeri* latex exhibited wound healing activity, possibly mediated by phytochemicals such as alkaloids in the aqueous fraction.

## Introduction

The genus *Jatropha* (Euphorbiaceae) comprises about 170 species of woody trees, shrubs, subshrubs, and herbs in the seasonally dry tropics of the Old and New World. Their stems, roots, leaves, seeds, fruits, and latex are used in medicinal folklore to treat various diseases [1]. However, some studies have warned against consuming them as herbal medicine; in particular, the seed oil can be highly toxic, and the leaves of *J. integerrima* can provoke vomiting and dehydration. For these reasons, their use in herbal medicine is cautioned against [2,3]. Nonetheless, *Jatropha* lattices are still widely used in medicinal folklore to treat ulcers and wounds [4,5].

In the past two decades of the 20^th^ century, several studies have reported on the beneficial properties of *Jatropha* lattices. Proanthocyanidin polymers have been found in the latex of *J. multifida* and showed anti-complement activity [6]. The latex of *Jatropha curcas* has a proteolytic enzyme of around 22 kDa, and the diluted latex showed healing action when applied topically, although the undiluted latex produced caustic lesions [7,8]. In addition, a novel cyclic nonapeptide with L-amino acids was identified in *J. podagrica* latex with cytotoxicity activity [9].

In the early 21^st^ century, some researchers continued to explore the potential uses of *Jatropha* lattice and their chemical characterizations. The whole *J. curcas* latex reduced the clotting time of blood, but its diluted latex prolonged it; this is significant because it demonstrates that the latex possesses both pro-hemostatic and anti-hemostatic activities [10]. In addition to *Jatropha*, the latex of other plants has also demonstrated thrombin-like proteases that can treat bleeding wounds [11]. The presence of cyclic nonapeptide in lattices supports their potential use as cytotoxic compounds against tumoral cells [12-14]. Other biological activities identified in lattices include anti-inflammatory effects and wound healing. The lattices of *Jatropha neopauciflora* Pax species, which are endemic to Mexico, promote wound healing that can attain up to 100% of the tensile strength of undamaged skin in a murine model. These lattices also promote antibacterial activity without toxicity when tested in cell lines [15]. When the cells infiltrating the lesion site were characterized, it was noted that CD68+ and CD34+ cells mobilized to skin tissues treated with *J. curcas* Linn. latex [16,17].

In Yucatán, Mexico, *Jatropha gaumeri* Greenm., an endemic tree, is commonly known in the Mayan language as *pomolché*, is commonly used to treat mouth blisters and skin rashes. In the previous studies, terpenoids with antimicrobial and antioxidant activity have been isolated from the crude extracts of *J. gaumeri* Greenm. roots and leaves, respectively [18,19]. A recent study found that the latex promoted analgesic and anti-inflammatory activity on buccal ulcers [20]; however, further experimental studies are needed to demonstrate and corroborate its biological activities, especially concerning the latex of the *J. gaumeri*.

This study aimed to demonstrate the wound healing activity of a cream formulation of *J. gaumer*i Greenm. latex in a murine model, provide a histological assessment of its scarring effects, and identify the family of phytochemicals involved in these effects.

## Materials and Methods

### Ethical approval

The experiments in this study were performed in adherence to the Mexican federal regulations for animal use and care (Nom-062-Zoo-1999) and under the institutionally approved protocol PE-2017-20 at the Autonomous Campeche State University.

### Study period and location

The latex sample and experimental study were carried out during the period between April 2018 to June 2019 in Campeche City, Mexico.

### Latex collection

*J. gaumeri* plants previously authenticated by the Biology Faculty of the Autonomous Campeche State University were grown in a backyard in a Campeche city neighborhood. *J. gaumeri* latex was obtained as a milky fluid exudate from cut stalk leaves and young stems and was stored in sterile tubes with protection from light. Previous asepsis was done with sterile water to ensure asepsis. The samples were transported in an icebox to the laboratory, kept at 4°C, and immediately fractioned.

### Latex extraction and cream formulation

A 25 mL volume of latex was suspended in a water-to-latex ratio of 1:2. The no aqueous phase was then partitioned successively with 2:1 chloroform, and the no chloroform phase partitioned 2:1 in ethyl acetate. Three fractions were obtained (aqueous, chloroform, and ethyl acetate). The aqueous fraction concentrated by lyophilization in small volumes of 5-10 ml in a 50 ml flask to optimize the process. The chloroform and ethyl acetate solvent elimination was carried under reduced pressure by rotavapor at 30-35°C. In addition, we used whole lyophilized latex, the dried powder was used to prepare 5% mixture in glycerin for experimental testing in mice to screen for wound healing activity. All extracts with wound healing activity were prepared further at 1% and 5% dilutions in carboxymethylcellulose and retested in the same murine model.

### Assessment of wound healing activity in a murine model

Fifty male Balb/c mice aged 10-12 weeks were obtained from the animal laboratory facilities of Research Center Biomedical Investigation, Autonomous Yucatan State University. The mice were divided into 10 experimental groups consisting of five mice each, randomized, and kept in separate cages in a well-ventilated temperature-controlled (24-27°C) environment with *ad libitum* access to a conventional diet and water. We adhered to the Mexican federal regulations for animal use and care (Nom-062-Zoo-1999).

The experimental groups were as follows: Group 1 consisted of healthy controls without wounds. The other nine groups had experimentally induced wounds treated with A-Derma (Avoine Rhealba, France) as a positive control (Group 2), glycerin as a placebo control (Group 3), 5% aqueous latex fraction in glycerin (Group 4), 5% ethyl acetate latex fraction in glycerin (Group 5), 5% chloroform latex fraction in glycerin (Group 6), 5% whole latex in glycerin (Group 7), 5% lyophilized latex in glycerin (Group 8), 1% aqueous latex fraction in carboxymethylcellulose (Group 9), or 5% aqueous latex fraction in carboxymethylcellulose (Group 10).

In this experiment, the mice were anesthetized, and an aseptic incision of 1 cm was made in a previously shaved area on the back. The wounds were topically treated every 24 h with the respective extracts for 22 days. The progression of wound healing was recorded at 1, 5, 10, 15, and 20 days. On day 22, the mice were euthanized, and the tensile strengths of the wounds were determined as previously described [19]. The tensile strength of the skin was defined as the amount of force in grams required to tear the skin. The percentage of wound healing efficacy was calculated as: % wound healing efficacy=(Tensile strength of scarred skin in grams/Tensile strength of healthy skin in grams)×100.

### Histopathological assessment of wounds

Skin tissue specimens were extracted from the wound sites and immediately fixed in 10% buffered formaldehyde, embedded in paraffin, sliced into sections 4 μm thick, and stained with hematoxylin-eosin and Masson’s trichrome.

### Chemical screening by thin-layer chromatography

The solvent system for polar extracts was ethyl acetate-methanol-water (100:13.5:10). The reagents used for detection were Dragendorff’s reagent, polyethylene glycol, and vanillin-sulfuric acid for alkaloids, flavonoids, and terpenes, respectively.

Three different samples were tested: Whole extract, chloroform latex fraction, and aqueous latex fraction. The ethyl acetate latex fraction was not analyzed because it did not have wound healing effects.

## Results

### Macroscopy observation of wound healing

The progression of wound healing in the mice was recorded at 1, 5, 10, 15, and 20 days. On day 5, the lesions remained open in all mice except in the positive controls treated with A-Derma^®^ and those treated with whole latex. White scars had formed in the positive control group, whereas dark scars had formed in the group treated with whole latex. On day 10, the lesions were dramatically reduced in the positive control and aqueous latex fraction groups. After 2 weeks, the lesions had completely closed, and regrowth of hair had begun in the positive control and aqueous latex fraction groups. Likewise, the mice treated with only glycerin exhibited closed lesions like those treated with the chloroform and ethyl acetate latex fractions. Notably, the lesions remained open in the mice treated with whole latex.

**Figure-1:**
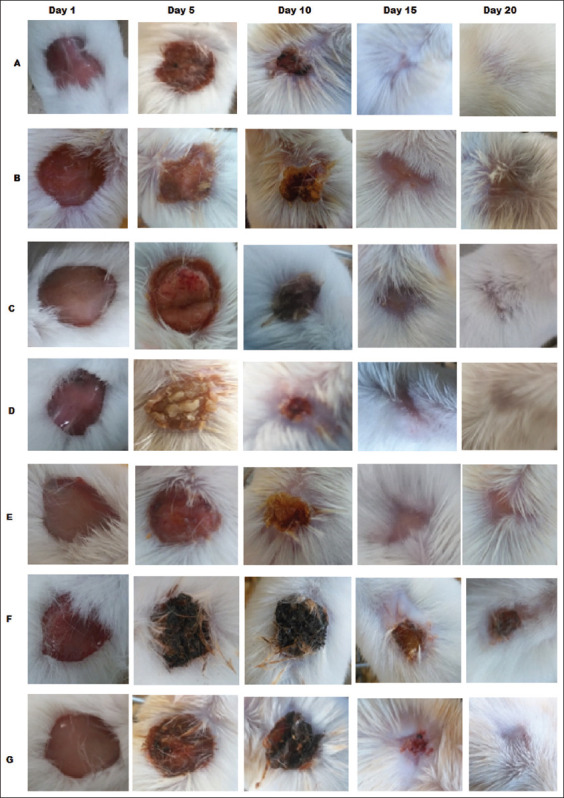
The visual aspect of the progression of wound healing. The wounds were topically treated every 24 h with the respective extracts for 22 days. The progression of wound healing in the mice was recorded at 1, 5, 10, 15, and 20 days.

During the last observation on day 20, wound healing was almost complete in the positive control and aqueous latex fraction groups. In the negative control (placebo) group treated with glycerin, the lesions were completely closed, as were the case for the chloroform and ethyl acetate latex fraction groups, but the hair regrowth was incomplete (Figure-1). These results indicate that the aqueous latex extract and the A-derma^®^ positive control promoted wound healing with similar efficacies.

### Wound healing efficacy (glycerin vehicle)

At the end of the experiment, and immediately after euthanized the mice; the tensile strengths of the wounds were measured, and the wound-healing efficacies were calculated. We found the highest wound healing efficacies in the aqueous latex fraction (97.7%) and chloroform latex fraction (93%) groups, which were higher than that of the positive control (79.5%). For the glycerin (negative control), ethyl acetate latex extract, and whole latex groups, the wound healing efficacy was between 72 and 77%. The lyophilized latex demonstrated the poorest overall wound healing efficacy (59%), which was worse even than that of the glycerin placebo (Table-1).

**Table-1:** Tensile strengths of the wounds using glycerin as vehicle.

Group	Force in grams	The percentage of wound healing (%)
Intact skin	220±5	100
Positive control	175±20	79.5
Placebo (glycerin)	160±15	72.7
5% aqueous latex fraction/glycerin	215±10	97.7
5% chloroform latex fraction/glycerin	205±5	93.1
5% ethyl acetate latex fraction/glycerin	160±10	72.7
5% whole latex/glycerin	170±10	77.2
5% lyophilized latex/glycerin	130±10	59

On the 22^nd^ day after treatment, the mice were euthanized, and the tensile strengths of the wounds were determined

### Wound healing efficacy (carboxymethylcellulose vehicle)

The tensile strengths of the wounds were measured in the second set of mice comprising two experimental groups (1% and 5% aqueous latex fraction in carboxymethylcellulose). The wound healing efficacies of each were 72% and 84%, respectively; these percentages closely resembled those of the placebo and positive control, but lower than that of the aqueous latex fraction in glycerin (Table-2).

**Table-2:** Tensile strengths of the wounds using carboxymethylcellulose as vehicle.

Group	Force in grams	The percentage of wound healing (%)
Intact skin	220±5	100
Positive control	175±20	79.5
1% aqueous latex fraction /carboxymethylcellulose	160±10	72.7
5% aqueous latex fraction/ carboxymethylcellulose	185±5	84
Placebo (carboxymethylcellulose)	160±15	72

On the 22^nd^ day after treatment, the mice were euthanized, and the tensile strengths of the wounds were determined

### Histology of skin samples

**Figure-2:**
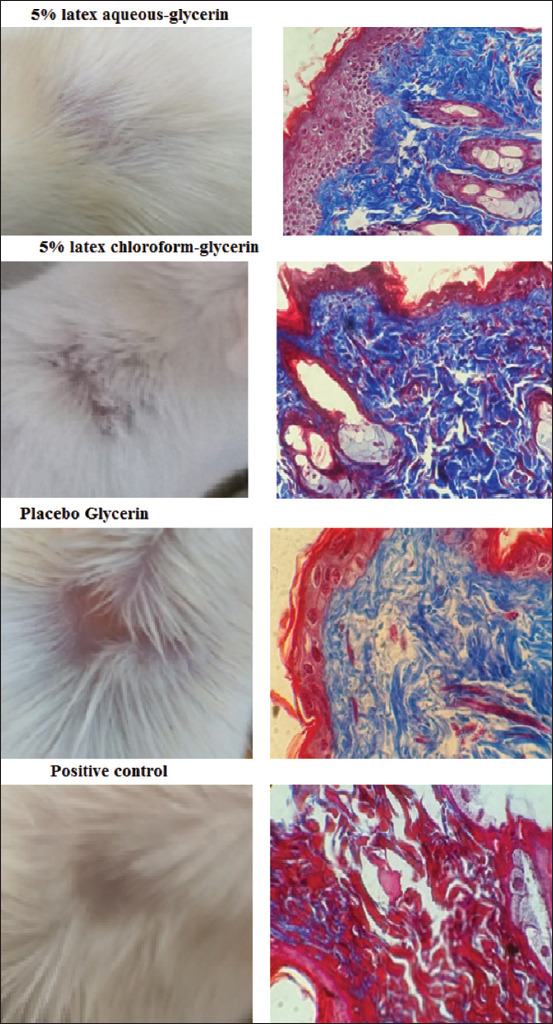
Histology findings in skin wound healing. Skin tissue specimens were extracted from the wound sites and immediately fixed in 10% buffered formaldehyde, embedded in paraffin, sliced into sections 4 μm thick, and stained with hematoxylin-eosin and Masson’s trichrome.

In untreated and healthy skin, we observed that the collagen fibers beneath the epidermis were compact, well-organized, and wavy, with moderate cellularity, well-conserved hair follicles, and sebaceous glands in the deep dermis. Conversely, in the skin of the positive control mice treated with A-Derma^®^, collagen was less abundant and organized into thicker fibers, whereas the cellular cytoplasm had strong red staining, presumably due to the activation and presence of myofibroblasts. In addition, the hair follicles had recovered. In the placebo group treated with glycerin, the appearance of collagen resembled that of normal skin except for reduced cellularity in the dermis, but we noted that the hair follicles were less evident than in the positive control and untreated skin. In the group treated with 5% aqueous latex fraction in carboxymethylcellulose, collagen fibers were less abundant, but hair follicles were present. Finally, the epidermis, and the dermis displayed organized reepithelialization with hair follicles and glands such that it resembled normal skin in groups treated with 5% aqueous latex fraction or 5% chloroform latex fraction in glycerin, (Figure-2).

### Chemical screening by thin-layer chromatography

**Figure-3:**
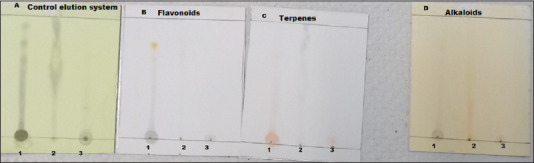
Thin-layer chromatography. Lane 1 whole extract, lane 2 chloroform latex fraction, and lane 3 aqueous latex fraction revealed with polyethylene glycol, Dragendorff’s reagent, and vanillin-sulfuric acid for flavonoids, terpenes, and alkaloids, respectively.

Thin-layer chromatography analysis revealed the presence of flavonoids, alkaloids, and terpenes in whole latex. When we analyzed the fractions separately, terpenes and alkaloids were detected in the aqueous fraction, while in the chloroform fraction had terpenes but not flavonoids or alkaloids (Figure-3).

## Discussion

Wound healing depends on the intricate and complex collaborative activity of several cell types, including keratinocytes, fibroblasts, endothelial cells, immune cells, white fat cells, and tissue-specific stem cells. Reticular fibroblasts move from deeper layers of the skin to initiate reepithelialization, thereby producing thick well-organized collagen fibers, whereas papillary fibroblasts stimulate new hair follicles by inhibiting Wnt/β-catenin signaling and consequent fibrosis [21]. In addition, dermal white adipose tissue and adipogenesis can help recruit fibroblasts to the wound site, favoring wound closure, and the regeneration of hair follicles. Finally, myofibroblasts, contractile collagen-secreting cells, are activated during the wound healing process, and their precursor cells comprise fibroblasts, endothelial, and epithelial cells. Excessive stimulation can result in fibrosis, while insufficient stimulation can delay wound healing [22].

In this context, we macroscopically observed that mice treated with aqueous latex fraction and A-Derma^®^ (positive control) had similar rates of healing and extents of wound closure, but hair growth was more abundant in the aqueous latex fraction group than in the positive control. The other latex extracts, as well as whole unfractionated latex, exhibited weaker efficacy in wound healing. These data suggest that phytochemicals with wound healing activity are more abundant and concentrated in the aqueous fraction.

In a previous study, we found that *J. gaumeri* latex demonstrated analgesic and anti-inflammatory activity in patients with buccal ulcers [20]. We have now also demonstrated the wound healing effects of *J. gaumeri* latex in a murine model. To date, the phytochemical composition of *J. gaumeri* latex and its medicinal activities have not been extensively studied; however, several published papers have demonstrated the antimicrobial, anti-inflammatory, wound healing, angiogenic, coagulant, and anticoagulant effects of other *Jatropha* species [6-8,10,11,16,17,23]. There is no doubt that the lattices of *Jatropha* species have broad spectra of activity that deserves further research to elucidate the active phytochemicals involved.

The wound healing and reepithelialization at the histological level in mice treated with aqueous latex fraction produced results that closely resembled that of healthy skin in terms of the appearance of collagen, the regeneration of hair follicles, and cellularity in the dermis, which showed organized epithelialization. On the other hand, the positive control and placebo groups had wound sites that resembled healthy skin but with less collagen organization and progression of new hair follicles. In addition, whole latex and the ethyl acetate latex fraction resulted in delayed wound healing, and reepithelialization was still in progress at the end of the experiment. We made the significant observation that mice treated with extracts in glycerin instead of carboxymethylcellulose showed improved wound healing, which indicates that the choice of vehicle used may influence wound healing.

It should be noted that Masson’s trichrome is limited in its ability to reveal the complexity of regeneration of injured skin at the cellular and molecular levels; however, hypertrophic or keloid scars were not observed in any treated group. Nonetheless, it is necessary to use other tools, such as histochemistry and molecular techniques, to elucidate the mechanism of action of the latex’s wound healing effects. Although there are few reports on the mechanism of action of latex, two papers have recently described the infiltration and mobilization of CD68+ and CD34+ cells in skin tissues treated with *J. curcas* Linn. latex [16,17].

Wound healing efficacy was assessed by mechanically measuring the tensile strength of the wound and expressing it as a percentage of that of healthy skin. We observed that the wound healing efficacy in mice treated with the aqueous latex fraction was 97%, similar to that of healthy skin; these data agree with the histologic and macroscopic observations. However, in mice treated with the A-Derma^®^ positive control, the wound healing efficacy was 79%. Both the aqueous latex fraction and positive control groups appeared to have identical macroscopic features, but on further analysis, the aqueous latex fraction yielded better results. The mice treated with whole latex and ethyl acetate latex extract experienced the worst results: 59% and 72% wound healing efficacy, respectively. These data parallel the histological analysis and progress of reepithelialization.

Significantly, whole latex was less effective in promoting wound healing than 5% aqueous latex fraction; these data may suggest that the concentration and composition of phytochemical compounds are an important factor in promoting specific activity, as reported in a study where whole undiluted *J. curcas* latex shortened the clotting time of blood but diluted latex prolonged it [10]. Similarly, diluted *J. curcas* latex has been shown to have healing properties, but undiluted latex has produced caustic lesions [8]. These findings contrast with another study in which the latex of *J. neopauciflora* Pax showed wound healing efficacy at 50%, 70%, and even 100% concentrations [15]; this contrasts with the data of Salas *et al*. who reported caustic lesions [8] and our results. In summary, the above data suggest that the composition and concentration of phytochemicals can differ significantly among the latex of different *Jatropha* species.

Finally, the composition analysis of the latex and its fractions revealed flavonoids, alkaloids, and terpenes in whole latex, while terpenes and alkaloids were detected in the aqueous fraction, which exhibited the best wound healing activity. Conversely, only terpenes were found in the chloroform fraction, which had poorer wound healing activity. According to this broad analysis, it is likely that alkaloids were the phytochemical compounds responsible for wound healing activity because they were not detected in the chloroform fraction. Although the composition of latex is similar among *Jatropha* species, some phytochemicals are present at lower concentrations or completely absent depending on the specific *Jatropha* species [10,15,24,25]. The differences in latex phytochemical composition detected among different published papers may suggest that composition is dependent on the species of *Jatropha* species and the sensitivity of the analytical techniques used.

## Conclusion

*J. gaumeri* latex exhibited wound healing activity in its aqueous fraction, with alkaloids likely being the phytochemicals involved. When used in an experimental model of wound healing, the resulting collagen appearance, hair follicle density, and dermis cellularity resembled normal healthy skin with organized epithelialization and without hypertrophy or keloid structures.

## Authors’ Contributions

VM and FL conceived and designed the study. GM, JAL, and VH collected all samples. VM and FL wrote and revised the manuscript. JAL,VH, and GM performed data analysis and MCB performed the examinations. All authors read, edited, and approved the final manuscript.
